# Molecular mechanisms of mucormycosis—The bitter and the sweet

**DOI:** 10.1371/journal.ppat.1006408

**Published:** 2017-08-03

**Authors:** Clara Baldin, Ashraf S. Ibrahim

**Affiliations:** 1 The Division of Infectious Diseases, Los Angeles Biomedical Research Institute at Harbor, University of California Los Angeles (UCLA) Medical Center and the St. John’s Cardiovascular Research Center, Torrance, California, United States of America; 2 Department of Medicine, David Geffen School of Medicine at UCLA, Los Angeles, California, United States of America; McGill University, CANADA

## Introduction

During the past 2 decades, mucormycosis has become the third most common invasive fungal infection in patients with hematological malignancies and organ transplantations [1]. This life-threatening disease is caused by ubiquitous fungi in the order Mucorales, predominantly by *Rhizopus* species including *R*. *delemar* and *R*. *oryzae*. Other common causative organisms include species of *Mucor*, *Lichtheimia* (previously *Absidia*), *Apophysomyces*, *Rhizomucor*, and *Cunninghamella* [1, 2]. The main risk factors for developing mucormycosis are neutropenia due to cancer treatment, hematopoietic and solid organ transplantation, diabetes mellitus, in particular when presenting with ketoacidosis (DKA), and other forms of acidosis. However, immunocompetent subjects can be affected when afflicted with trauma (e.g., soldiers in combat operations and patients with injuries due to natural disasters) [3, 4]. It is likely that mucormycosis will continue to increase in incidence because the number of organ transplantations, cancer patients, and diabetic patients is on the rise. Just in the United States in 2015, the number of transplant patients exceeded 30,000, with an increase of nearly 5% over 2014 (Organ Procurement and Transplantation Network [OPTN]). According to WHO, the number of people affected by diabetes quadrupled in the past 4 decades, reaching >420 million in 2014. Additionally, due to global warming, natural disasters with outbreaks of mucormycosis are likely to occur with higher frequency, similar to what happened with the 2004 Southeast Asia tsunami [5] and the 2011 Joplin tornado [4].

Despite current treatment options, which often include widespread disfiguring surgical intervention and antifungal therapy, mortality rates due to mucormycosis range between 50%–100% [6]. Consequently, novel strategies to prevent and/or treat mucormycosis are needed and can be facilitated by understanding the pathogenesis of the disease.

## Clinical features predict distinctive pathogenicity traits

Mucorales can gain entry to a susceptible host through inhalation, ingestion of contaminated food, and through an abraded skin. These routes result in rhinoorbital/cerebral, pulmonary, gastrointestinal, or cutaneous infections [1]. Regardless of the manifestation of the disease, a hallmark of mucormycosis is the ability of the causative organism to aggressively and rapidly invade blood vessels, which results in hematogenous dissemination, vessel thrombosis, and subsequent tissue necrosis [1]. Therefore, interactions between invading fungi and endothelial cells lining blood vessels represent a major step in the pathogenesis of mucormycosis. Similarly, the unique predisposition of DKA patients and deferoxamine-treated patients to mucormycosis points to the importance of hyperglycemia, iron, and acidifying ketone bodies in the virulence of Mucorales. In this Pearl, we summarize recent advances in our knowledge of the effect of these factors on the virulence of Mucorales and modulation of the fungus interactions with endothelial cells.

## Mucorales invade the endothelium through unique receptors

Basement membranes are constituted of extracellular protein matrices that are mainly composed of laminin and collagen IV. These membranes separate epithelial or endothelial cells from underlying stroma [7]. Due to epithelial cell damage (e.g., due to diabetes or chemotherapy), the extracellular matrix proteins can be exposed for direct interaction with inhaled or ingested spores. It has been shown that *Rhizopus* spores adhere to laminin and type IV collagen [8]. The attachment of the fungal spores to extracellular protein matrices is specific, because antilaminin and anticollagen antibodies as well as receptor competition experiments block adhesion to extracellular proteins [8].

Although not much is known about how Mucorales interact with epithelial cells, *Rhizopus* adheres to and invades endothelial cells by specific recognition of the host receptor glucose-regulator protein 78 (GRP78) [9, 10]. This recognition causes host cellular death by induction of the endothelial cell–mediated fungus endocytosis. GRP78, which was first discovered as a heat shock protein involved in stress-related responses [11], binds to *R*. *delemar* as well as other Mucorales germlings but not spores [9]. Binding to germlings is consistent with the hypothesis that hyphae are the invading form of Mucorales. Similarly, induced endocytosis and active penetration of epithelial cells by *Candida albicans* is linked to the formation of hyphal structures, and mutants impaired in hyphal formation are defective in host cell invasion [12, 13]. *Aspergillus fumigatus*, instead, invades lung epithelial or endothelial cells by expressing the thaumatin-like protein CalA on both germlings and conidia [14]. Although an earlier study showed that CalA is required for adherence of *A*. *fumigatus* to laminin [15], hyphal adhesion to host cells and macromolecules was shown to be predominantly mediated by the polysaccharide galactosaminogalactan [16]. Suppression of GRP78 expression by short hairpin RNA (shRNA) or blocking its function by antibodies suppresses fungal invasion of host cells and drastically decreases endothelial cell injury caused by *R*. *delemar* but not that of other fungal pathogens like *C*. *albicans* or *A*. *fumigatus*. Importantly, anti-GRP78 antibodies protect DKA mice from mucormycosis [9].

The fact that GRP78 does not bind to *C*. *albicans* or *A*. *fumigatus* and anti-GRP78 antibodies do not affect endothelial cell invasion of these 2 fungi clearly shows that a unique mechanism of Mucorales-mediated endothelial cell invasion and injury exists [9]. Also, the lack of complete abrogation of *Rhizopus*-mediated endothelial cell invasion and injury when GRP78 is blocked or suppressed indicates that other factors are involved in *Rhizopus* interacting with endothelial cells. In this respect, in preliminary results of the transcriptome of endothelial cells interacting with *R*. *delemar*, *R*. *oryzae*, or *M*. *circinelloides*, it was shown that the platelet derived growth factor (PDGF) pathway is activated [17], similarly to *C*. *albicans* [18]. Indeed, the use of 2 small molecules that inhibit the phosphorylation of PDGF receptor partially reduces *Rhizopus*-mediated endothelial cell injury in vitro [17]. Future investigations are required to delineate whether GRP78 and PDGF receptor act as coreceptors or independently in facilitating Mucorales invasion of endothelial cells.

The fungal ligand that binds to GRP78 during invasion of the endothelium belongs to the spore coating (CotH) protein family. Similar to the previous discoveries for GRP78, blocking the function of CotH proteins either biochemically by using anti-CotH antibodies or genetically by attenuating *CotH* expression reduces the ability of *R*. *delemar* to invade and injure endothelial cells in vitro and reduces disease severity in mice [19]. CotH proteins are universally present in Mucorales and absent from any other forms of life for which the genome has been sequenced [19]. In other pathogenic fungi, invasion of host cells is mediated by other cell surface proteins. For example, agglutinin-like sequence (Als) proteins and thaumatin-like protein (CalA) have been reported to act as invasins for *C*. *albicans* and *A*. *fumigatus*, respectively [13, 14, 20]. All 3 classes of protein families are characterized by the presence of secretion-signal and glycophosphatidylinositol (GPI)-anchored sequences. However, they all bind to different host receptors (e.g., Als proteins bind to cadherins [20] while CalA binds to integrins [14]).

The most commonly isolated Mucorales from patients (*Rhizopus*, *Mucor*, and *Lichtheimia*) contain 3–7 copies of *CotH*, while those that are only occasionally the cause of the disease (*Apophysomyces*, *Cunninghamella*, *Saksenaea*, and *Syncephalastrum*) only contain 1–2 copies [17]. Interestingly, isolates of Entomopthorales, which were previously taxonomically considered close to Mucorales but do not cause invasive disease, lack the presence of *CotH* [17]. Collectively, these data point to the unique interaction between Mucorales CotH and endothelial cell GRP78 receptor and to the ability of CotH to mediate invasive disease. Moreover, it appears that Mucorales fungi harboring more copies of *CotH* can cause more frequent disease. Alternatively, it is possible that GRP78 among individuals harbors SNPs that make the attachment to certain sequences of CotH more avid compared to others. This assumption is supported by the fact that CotH2 and CotH3, which have 77% sequence identity, can avidly bind GRP78 while CotH1, which has about 20%–24% amino acid identity to CotH2 and CotH3, does not [19]. Future studies are necessary to verify these hypotheses and their effect on the interaction between GRP78/CotH and the frequency and severity of the disease [19].

## Glucose, iron, and acidosis by β-hydroxy butyrate modulate GRP78/CotH interactions

**Fig 1:**
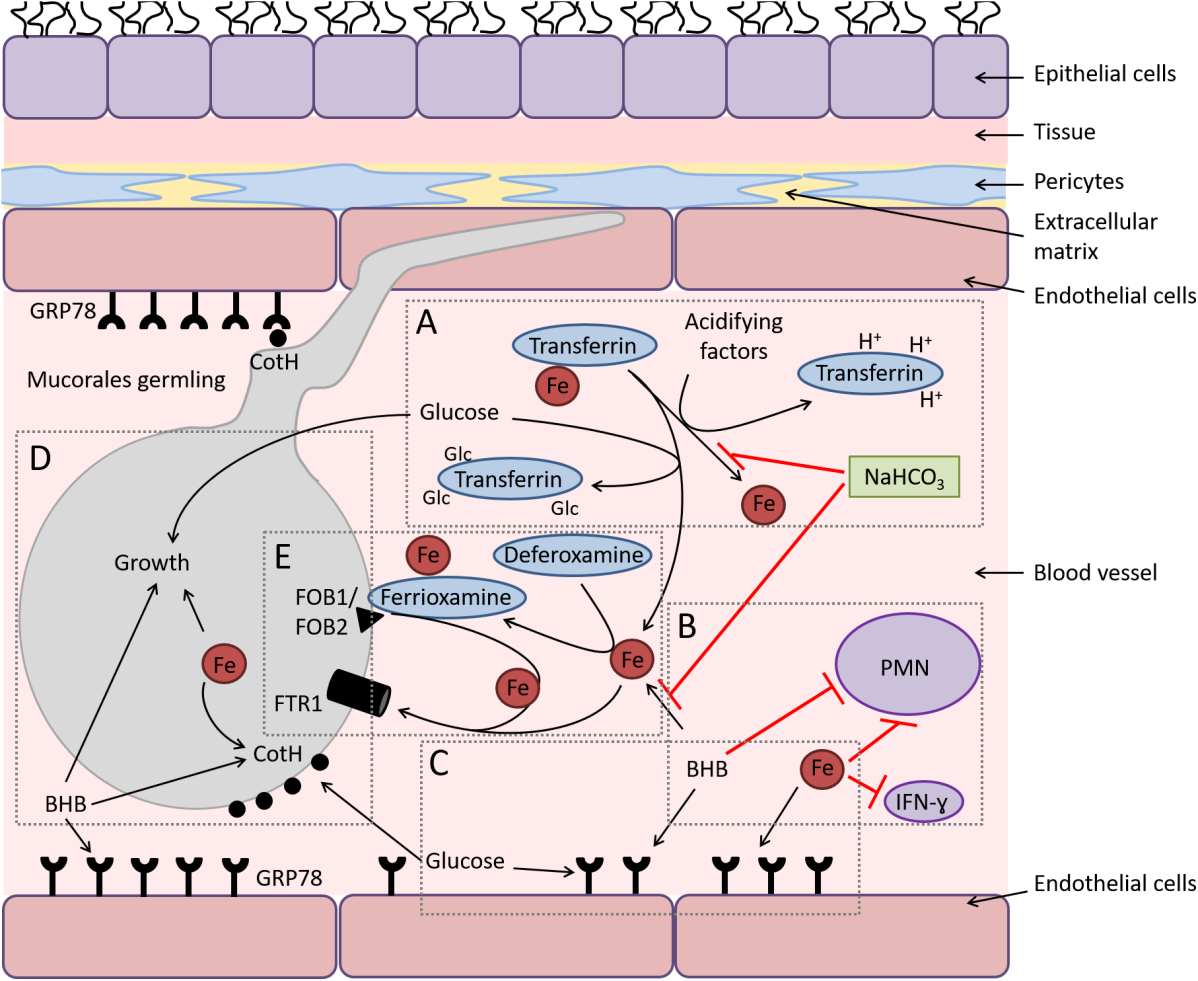
Diagram depicting the interactions of Mucorales with endothelial cells during hematogenous dissemination/organ seeding and the effect of host factors on these interactions and on the immune response. (A) Hyperglycemia and ketoacidosis result in liberation of iron from serum-sequestering proteins (e.g., transferrin) via glycosylation and protonation, respectively. (B) Ketone bodies (e.g., β-hydroxy butyrate [BHB]) and free iron negatively affect the immune response to the infection, while sodium bicarbonate (NaHCO_3_) reverses this negative effect by preventing iron release from transferrin and neutralizing acidity. (C) Surface expression of glucose-regulator protein 78 (GRP78) on endothelial cells is enhanced to cope with the stress elicited by hyperglycemia, free iron, and ketone bodies. (D) Glucose, free iron (transported by the high affinity iron permease [Ftr1p]), and BHB also enhance the expression of fungal cell surface CotH, which results in invasion of the endothelium and augmentation of fungal growth. (E) In deferoxamine-treated hosts, the iron-rich ferrioxamine binds to its fungal receptor (ferrioxamine binding proteins [Fob1/Fob2]) then releases iron via a reductive step prior to feeding invading Mucorales via Ftr1p transportation.

DKA and deferoxamine-treated patients are uniquely predisposed to mucormycosis. Clearly, diabetic patients suffer from an elevated concentration of glucose [1]. Hyperglycemia can induce excessive glycosylation of proteins such as transferrin and ferritin, diminishing their iron affinity [21]. Moreover, in the presence of an acidotic condition due to accumulation of ketone bodies (e.g., β-hydroxy butyrate [BHB]), the low pH in the blood vessels strongly impairs the ability of transferrin to chelate iron [22]. Glucose, iron, and BHB enhance the growth of the fungus (**Fig 1**) [23, 24]. They also induce the expression of GRP78 and CotH, and this enhanced expression results in augmented fungal invasion and subsequent injury of the endothelium in vitro (**Fig 1**) [9, 24]. It appears that the BHB-related acidosis exerts a direct effect on both GRP78 and CotH expression (an effect not seen with lactic acid) and an indirect effect by compromising the ability of transferrin to chelate iron, because iron chelation combined with reversal of pH by sodium bicarbonate completely protects endothelial cells from *Rhizopus*-mediated invasion and injury (**Fig 1**) [24]. Importantly, DKA mice, or those treated with BHB, suffer from lower blood pH, have elevated available serum iron, express more GRP78 in their target organs (e.g., lungs and sinuses), and are extremely susceptible to mucormycosis [9, 24]. Consistent with the clinical observation, ketoacidosis does not predispose mice to aspergillosis [24]. It is also worth noting that physiological concentrations of glucose, iron, and BHB augment the fungal growth and have detrimental effect on the host immune response via suppression of T-lymphocyte induction, interferon-Ɣ production, and phagocyte-mediated killing (**Fig 1**) [24–28]. Thus, the unique interactions of GRP78 and CotH proteins and their enhanced expression under hyperglycemia and ketoacidosis explain the specific susceptibility of DKA patients to mucormycosis.

To emphasize the importance of GRP78/CotH protein interactions in the pathogenesis of mucormycosis, therapeutic treatment with either anti-GRP78 or anti-CotH antibodies protect DKA and neutropenic mice from mucormycosis [9, 19, 29]. Also and potentially of clinical relevance is the finding that reversal of ketoacidosis in *Rhizopus*-infected mice by administration of sodium bicarbonate (in lieu of insulin) improves survival [24]. This protection is believed to be caused by reversal of the enhanced fungal growth, restoration of the immune function, and halting of host tissues fungal invasion. It is currently unknown what role the GRP78/CotH interactions play in the neutropenic host, the other major patient population susceptible to mucormycosis. However, the fact that anti-CotH antibodies protect cyclophosphamide/cortisone acetate–treated mice from Mucorales infections [29] argues that at least CotH proteins play a major role in the virulence of Mucorales in this host. Also, it has been known that GRP78 expression induces resistance of cancer cells to chemotherapy [30]. Therefore, it is possible that cyclophosphamide treatment results in induction of GRP78 expression in mice.

## Other factors contributing to endothelium injury

The recognition of CotH by GRP78 does not appear to be the only mechanism through which Mucorales damage endothelial cells. It was observed that nonviable *Rhizopus*, killed by heat or chemicals such as glutaraldehyde or ethanol, was able to cause a comparable amount of damage to endothelial cells as viable cells [10]. These results suggest the contribution of toxin-like substances in mucormycosis pathogenesis. However, rhizotoxin, which is produced by the *Rhizopus* symbiont bacterium *Burkholderia*, does not contribute to the virulence of *Rhizopus* [31–33]. Therefore, it is reasonable to speculate the presence of toxin-like secondary metabolites produced directly from Mucorales, which mediate the interaction between the pathogen and the host, especially with the recent report showing *M*. *circinelloides* to be the cause of food poisoning illness [34]. Genomic evidence also supports the notion that Mucorales possess pathways for secondary metabolites including polyketide synthases (PKSs), nonribosomal peptide synthetases (NRPs), and L-tryptophan dimethylallyl transferases (DMATs) [35].

## Host iron acquisition is essential for establishing infection

As mentioned above, hemodialysis patients receiving the bacterial siderophore deferoxamine for treating iron overload are uniquely predisposed to highly lethal and frequently disseminated mucormycosis [36, 37]. Also, administration of deferoxamine to animals increases their susceptibility to mucormycosis [38, 39]. Although deferoxamine prevents iron-overload toxicity via efficiently chelating iron from the host, it is known that *Rhizopus* possesses cell-surface receptors to ferrioxamine (the ferric-rich form of deferoxamine) [36, 40]. These clinical and experimental observations highlight the importance of host iron acquisition in mucormycosis pathogenesis by supporting fungal growth during host cell invasion. It is currently unknown if deferoxamine-treated hosts suffer from elevated expression of GRP78. However, given the main function of GRP78 as a stress-related protein and the clinical observation that deferoxamine-treated patients usually suffer from disseminated disease, it is highly likely that GRP78 is overexpressed on endothelial cells to alleviate cell toxicity due to excess iron availability.

Emphasizing the importance of iron in mucormycosis pathogenesis is the improved survival and reduced tissue fungal burden of DKA mice treated with the FDA-approved iron chelator deferasirox, especially when used with the antifungal agent liposomal amphotericin B [41]. Similarly, case reports, particularly in diabetic hosts, showed beneficial outcome when deferasirox is used as adjunctive therapy [42]. However, a phase II clinical trial, mainly conducted in neutropenic patients with active malignancies, demonstrated that adjunctive deferasirox therapy is associated with increased mortality rate [43]. It is worth noting that this trial enrolled a small number of patients (a total of 20 patients in both arms) and suffered from major imbalances, with patients in the deferasirox arm more likely than placebo patients to have active malignancies, neutropenia, or corticosteroid therapy.

The ferrioxamine inducible receptors (ferrioxamine binding proteins [Fob1/Fob2]) [44] were recently identified to belong to the cystathionine beta synthase (CBS)-domain protein family, which is found in all kingdoms of life [45]. Although a study demonstrated that ferrioxamine is taken up entirely by *R*. *oryzae* via a siderophore shuttle mechanism [46], it is more likely that a major mechanism of iron uptake involves the release of iron from the siderophore prior to its transport intracellularly (**Fig 1**). This assumption is supported by 2 studies showing that iron uptake from ferrioxamine by *Rhizopus* is an energy-dependent process that requires reductase activity to convert the insoluble ferric into more soluble ferrous iron [40, 44]. Additionally, the use of the extracellular membrane–impermeable ferrous chelator bathophenanthroline disulfonate inhibits growth of *Rhizopus* when ferrioxamine is used as a sole source of iron, and this inhibition was due to attenuation of iron uptake from ferrioxamine [44]. Finally, *R*. *delemar* mutants with attenuated expression of the high-affinity iron permease (Ftr1, a cell membrane protein required to transport iron intracellularly [23]) have impaired ability to take up iron from ferrioxamine, experience retarded growth on media supplemented with ferrioxamine as a sole source of iron, and demonstrate reduced virulence in mice given deferoxamine [44, 47]. These mutants, however, do not have full abrogation of iron uptake from ferroxiamine, and their attenuated virulence in deferoxamine-treated mice is not complete. Thus, it is probable that the reductase/permease pathway represents a major uptake pathway for iron acquisition from ferrioxamine without internalization of the generated deferoxamine, while the uptake of the siderophore by shuttle mechanism [46] represents a secondary mechanism.

In a DKA host, the released iron from transferrin due to hyperglycemia and acidification of the milieu is also transported by the Ftr1p (**Fig 1**) because *R*. *delemar* mutants with reduced expression of Ftr1p have defective virulence in DKA mice [47]. Other potential mechanisms to sequester iron from the host rely on siderophores (rhizoferrins are siderophores synthesized by Mucorales [40, 48]) and heme oxygenase [49], but their role in the pathogenesis remains unknown.

## Conclusions and perspective

Mucormycosis angioinvasion is reliant on unique interaction between Mucorales CotH and endothelium GRP78, which triggers host cell injury and subsequent hematogenous dissemination of the fungus. DKA and deferoxamine-treated patients are predisposed to mucormycosis because elevated levels of serum glucose, iron, and BHB augment fungal growth and enhance the expression of GRP78 and CotH, which results in increased ability of Mucorales to invade host tissues. Strategies targeting CotH/GRP78 interactions are likely to prove beneficial in caring for patients with mucormycosis as adjunctive therapies. Other factors (e.g., mycotoxins) are also likely to be operative during infection and could be affected by conditions found in susceptible hosts.
